# New technologies in minimally invasive surgery in childhood cancer surgery

**DOI:** 10.3332/ecancer.2025.2029

**Published:** 2025-11-13

**Authors:** Abdelhafeez H Abdelhafeez, Sabine Sarnacki, Blanc Thomas

**Affiliations:** 1 University of Rochester Medical Center, Golisano Children's Hospital, Department of Surgery, Division of Pediatric Surgery, Rochester, NY 14642, USA; 2 Department of Paediatric Surgery, Urology, and Transplantation, Necker Enfants Malades Hospital, APHP and Paris Cité University, Paris 75015, France

**Keywords:** minimally invasive surgery, paediatric cancer, robotics, single-site surgery, image-guided surgery, surgical precision, tumour resection

## Abstract

**Objective:**

The aim of this guidance is to discuss the advantages of utilising adjunct technologies in minimally invasive surgery and to mitigate risks associated with these technologies in paediatric cancer surgery.

**Methods:**

A literature search was conducted, focusing on robotics, single-site and image-guided surgical approaches in paediatric cancer.

**Results:**

The findings indicate significant improvements in surgical precision, reduced morbidity and enhanced recovery times. Technologies such as robotics, single-site and image-guided surgical approaches have shown promising results in improving the precision of tumour resection.

**Conclusion:**

Integrating advanced technologies into paediatric cancer surgery offers the potential for improved surgical outcomes and quality of life for patients. However, ongoing research and careful implementation are necessary to ensure safety and efficacy.

## Introduction

Surgical resection is a cornerstone of the management of childhood solid tumours due to its critical role in achieving local control and improving survival outcomes [1–3]. Advances in treatment have highlighted the importance of minimally invasive surgical techniques, which aim to reduce the physical and psychological burdens of surgery [4].

Conventional laparoscopic surgery has been widely utilised in paediatric oncology for procedures such as tumour biopsy and resection [5, 6]. Laparoscopic approach typically involves multiple small ports, allowing for improved visualisation and reduced recovery times compared to open surgery. Despite these benefits, traditional laparoscopic approaches can have limitations, including instrument maneuverability, reduced depth perception and increased surgeon fatigue due to ergonomics. Additionally, the complexity of paediatric anatomy poses unique challenges in accessing tumours, particularly in the retroperitoneum of young children [5, 6]. Moreover, incomplete tumour resection remained a challenge since, for decades, intraoperative tumour identification relied on white light visualisation and tactile feedback.

The aim of this review is to discuss new technologies applications in paediatric cancer surgery, aiding more precise maneuverability, less tissue trauma and advanced tumour identification technologies such as robotic, single-site retroperitoneoscopic and image-guided surgical approaches, respectively.

## Robotic-assisted surgery

The application of robotic surgical systems in paediatric surgery has been limited by various intrinsic and extrinsic factors. However, their integration into the surgical management of solid tumours in children presents both unique challenges and significant opportunities. The gradual adoption of robotic technology in paediatric centers is poised to bring about a paradigm shift in surgical care, improving precision of movement and ergonomics [7].

A recent experience from a tertiary paediatric center demonstrated that oncology accounted for 23% of the total robotic surgical procedures since the program's inception. Within the oncology workload, 25% of tumours were treated using a robotic platform [8]. The median age of patients undergoing robotic-assisted surgery (RAS) was 7 years, with one-third of patients aged <5 years, one-third between 5 and 11 years and one-third >11 years. The most common tumour types included endocrine tumours (31%), neuroplastic tumours (29%) and renal tumours (19%). Notably, complication and conversion rates were minimal, underscoring the safety and efficacy of the robotic approach [8].

RAS offers enhanced precision, particularly in the delicate dissection of tumours and in situations where bleeding control is critical. However, the decision to use the robotic platform should be guided by factors such as tumour characteristics, preoperative imaging, tumour extent and the surgeon’s experience. Further research and the development of clearer, evidence-based guidelines are needed to refine its application across a broader range of paediatric solid tumours [8].

Contraindications for RAS in paediatric patients include neurogenic tumours with midline major vessels encasement, extension to the middle mediastinum (such as the pericardium, esophagus or trachea), as well as those involving more than two International Neuroblastoma Risk Factor Classification risk factors or involving unpaired vessels (e.g., the celiac artery or superior mesenteric artery) and/or both renal pedicles. Other contraindications include renal tumours crossing the median sagittal plane or tumours that invade the liver. Robotic surgery is also contraindicated for adrenocortical carcinomas and solid pseudopapillary tumours. Each indication of RAS must be validated by the multidisciplinary tumour board (oncologist, surgeon and radiologist) [8, 9].

**Figure 1.**
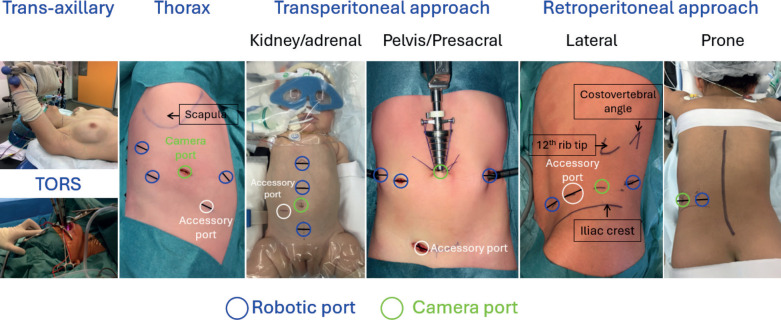
Anatomical sites of tumours and corresponding robotic resection camera and port approaches.

Three factors that may influence morbidity in robotic surgery have been analysed [10]. First, small patients (≤15 kg) have been identified as a potential challenge for robotic-assisted laparoscopic surgery due to limited working space [11]. Second, an American Society of Anesthesiologists (ASA) score of ≥3 is used as a marker of patient vulnerability, as it has been associated with increased perioperative morbidity from both surgical and anaesthetic factors [12, 13]. Third, surgical oncology was examined as a potential contributor to patient morbidity, given the complexity of procedures involving limited tumour exposure, the impact of preoperative chemotherapy on tissue dissection, potential multiorgan involvement, the risk of significant intraoperative bleeding and the oncological risks associated with tumour spillage or insufficient surgical margins [14]. In a recent study, none of these factors were linked to postoperative complications, although major complications (Clavien-Dindo ≥ III) may be more common in patients with a higher ASA score [10]. Figure 1 illustrates the locations of the robotic camera and ports for various tumour anatomies.

### Single-site surgery

**Figure 2.**
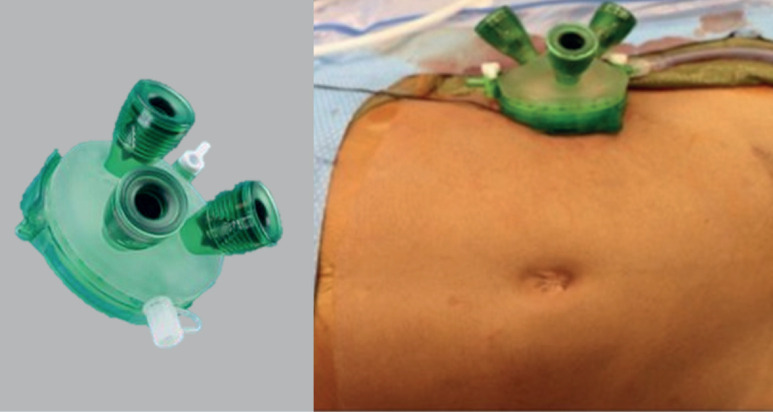
Single-site retroperitoneoscopic approach.

Single-site surgery has gained attention as a minimally invasive technique in paediatric oncology, particularly for the resection of tumours located in the retroperitoneum. This approach involves making a single small incision through which multiple ports provide access to the retroperitoneal, intraperitoneal or thoracic spaces for tumour removal [15–18] (Figure 2). The main advantage of single-site retroperitoneoscopic surgery is its ability to offer enhanced precision and maneuverability in the confined retroperitoneal space, allowing for careful and meticulous dissection [15–18]. Additionally, the single incision provides a relatively larger working port, which can also be used for specimen retrieval [15–18].

One key benefit of single-site retroperitoneoscopic surgery is faster access to control bleeding and potentially easier conversion into an open procedure if necessary [15–17]. Because the single-site incision is typically larger than those used in multiport access and is more directly aligned with the tumour site, it allows for a more straightforward transition to an open approach if complications occur.

For paediatric patients, this technique offers significant advantages, including reduced scarring, less postoperative pain and faster recovery times; factors that are especially important in children undergoing cancer treatment who benefit from quicker resumption of chemotherapy postoperatively [15–17]. Research has shown that single-site thoracoscopic surgery can be comparable to multiport thoracoscopic surgery in terms of postoperative pain and hospital stay [19]. Interestingly, the advantage of single-site retroperitoneoscopic surgery over conventional laparoscopic surgery may stem more from the retroperitoneal approach itself—avoiding entry into the peritoneal cavity and the need to mobilise bowel—rather than the use of a single site per se [15–17].

However, despite these benefits, the application of single-site surgery in paediatric oncology presents certain challenges. The learning curve to master the retroperitoneal approach, the complexity of retroperitoneal tumour resections, especially for large tumours or those involving critical structures like the renal vasculature, can limit the technique's applicability. Moreover, the process of creating the retroperitoneal space can sometimes result in unintentional breaches of the peritoneal cavity, causing it to collapse and reducing the available working space [20]. In such cases, conversion to laparoscopic access may be required. While peritoneal breaches can be repaired, this is technically challenging during retroperitoneoscopic surgery.

The success of this technique also depends heavily on the surgeon's experience with single-site retroperitoneoscopy and the availability of specialised equipment. Given these challenges, while the technique holds promise, further research and clinical experience are necessary to refine its application and establish comprehensive guidelines for its use in paediatric cancer surgery. Currently, applications for a single-site approach in paediatric cancers include resection of retroperitoneal primary and metastatic tumours, retroperitoneal staging template lymphatic dissection, nephron-sparing resection, resection of mediastinal tumours and resection of pulmonary metastatic nodules.

The launch of the single-port robotic system is a new frontier combining two advanced surgical approaches that would further enhance the precision of childhood cancer surgery [21].

### Image-guidance surgery

Image-guided surgery is increasingly recognised as a transformative tool in the treatment of paediatric cancers, potentially offering greater precision in primary tumour and metastatic deposits identification [22–32]. This technique includes several imaging modalities, with fluorescence-guided surgery and navigation imaging standing out as two of the most promising [22–34]. Fluorescence-guided surgery relies on the use of fluorescent agents that either passively or actively target tumour tissues [25–30]. Passive targeting agents, such as indocyanine green (ICG), are highly sensitive and can highlight tumour areas effectively. However, their specificity is often limited, which can sometimes result in false positives or difficulty distinguishing tumour from surrounding healthy tissue [25–30]. On the other hand, active targeting agents, often coupled with tumour-specific antibodies or receptors, are being explored in paediatric cancers but are still in the early stages of development. Their efficiency and clinical applicability remain to be fully evaluated [22, 24–30].

Augmented reality (AR) and navigation systems are also playing an increasingly important role in enhancing tumour localisation and improving surgeons’ understanding of tumour anatomy before (during the planning phase) and during surgery [22–24]. These technologies allow for the overlay of preoperative imaging data onto the surgical field in real time, creating an intuitive visualisation of tumour structures. Together, AR and navigation systems provide an additional layer of precision, potentially improving the likelihood of complete tumour resection while preserving healthy tissues, which is especially critical in paediatric patients.

In the context of paediatric cancers, ultrasound-guided tumour resection is emerging as a particularly promising tool. High-resolution ultrasound has shown great potential in liver surgeries and nephron-sparing procedures for bilateral Wilms tumour, allowing for localisation of deep tumour nodules [25, 26]. The application of such high-resolution ultrasound could be extended to other paediatric tumours, particularly in soft tissue tumours and sarcomas, where real-time visualisation of tumour boundaries is critical.

While many of these technologies are still evolving and require further refinement and validation, their combined use in paediatric cancer surgeries is paving the way for less invasive, more effective treatments, with the potential for better outcomes and reduced long-term effects for young patients.

## Discussion

The advent of advanced technologies such as robotic surgery, single-site surgery and image-guided surgical approaches is potentially transforming the landscape of paediatric cancer surgery. These technologies may offer improvements in surgical precision, which is a crucial consideration when treating paediatric cancer patients. Minimally invasive techniques, bolstered by these adjunct technologies, present promising opportunities to improve surgical outcomes and minimise the long-term physical and psychological burdens traditionally associated with open surgery.

Robotic surgery offers advantages in terms of dexterity, precision and visualisation. The ability to perform complex dissection with 3D imaging and articulated instruments enables surgeons to navigate complex anatomical structures with greater ease, which is particularly beneficial in the paediatric population, where working space is limited and vascular involvement is not uncommon. The enhanced visualisation and fine motor control provided by robotic systems are invaluable in paediatric oncology, where precision is critical, especially for tumours located near vital structures and major blood vessels.

However, the integration of robotic surgery into paediatric cancer treatment is not without challenges. The limited availability of robotic systems and the high cost of these technologies remain significant barriers to widespread adoption, particularly in resource-limited settings. Additionally, robotic surgery requires specialised training and it may not be suitable for all tumour types or locations, as certain tumours may pose challenges in terms of access or require more extensive resection than robotic systems can currently accommodate. Despite these challenges, the potential benefits in terms of precision and reduced morbidity make robotic surgery an exciting option for paediatric oncologic procedures and warren outcome comparative studies.

Single-site surgery, which involves performing procedures through a single small incision, is slowly gaining traction as a minimally invasive technique in paediatric oncology. One of its key advantages is the reduced scarring compared to traditional multiport laparoscopy, which can improve cosmetic outcomes, an important consideration in paediatric patients. Additionally, the use of a single incision can potentially minimise postoperative pain and expedite recovery, allowing for faster resumption of chemotherapy or other treatments [15–17].

Single-site retroperitoneoscopic surgery has shown promise for tumour resections in the retroperitoneal space, such as neuroblastomas, other neurogenic tumours, metastatic lymphadenopathy and staging lymph nodes sampling [15–17]. The main advantage of single-site retroperitoneoscopic surgery is the ability to offer greater precision and maneuverability in confined anatomical spaces. Moreover, the relatively larger working port provided by the single incision facilitates specimen retrieval and bleeding control, which can be more challenging with multiport access. This approach can also allow for easier conversion to an open procedure in retroperitoneal surgery if complications arise, since the incision is generally more directly oriented toward the tumour site.

However, the applicability of single-site surgery is not without limitations. The complexity of paediatric retroperitoneal surgeries, especially when dealing with large tumours or tumours involving critical structures such as vessels, can make this approach challenging. The risk of unintentional breaches into the peritoneal cavity during the creation of the retroperitoneal space is another concern, as it can reduce the available working space and necessitate conversion to laparoscopic or open surgery [19]. As with robotic surgery, the surgeon’s experience and expertise play a critical role in determining whether single-site surgery is a viable option for a given patient. More research is needed to refine this technique and establish best practice guidelines for its use in paediatric oncology.

Image-guided surgery is becoming increasingly important in paediatric oncology, offering the potential for preoperative planning, real-time tumour localisation and enhanced surgical precision. Fluorescence-guided surgery, for instance, has shown promise in localisation of tumour and metastatic deposits, which is particularly valuable in cases where traditional white light visualisation may be insufficient [26]. Fluorescent agents like ICG can highlight tumour areas with high sensitivity, though their specificity remains limited, making it challenging to clearly distinguish between tumour and surrounding healthy tissue [26]. The development of active targeting agents, such as those coupled with tumour-specific antibodies, holds promise for increasing the specificity and accuracy of fluorescence-guided surgery, though these agents are still in the early stages of clinical evaluation in paediatric cancers [22].

In addition to fluorescence guidance, other image-guided techniques such as AR and intraoperative navigation are increasingly being incorporated into paediatric cancer surgery. These technologies allow surgeons to overlay preoperative imaging data onto the surgical field in real time, potentially enhancing tumour localisation and improving the accuracy of resection.

Ultrasound-guided surgery has been particularly useful in liver surgeries and nephron-sparing procedures for bilateral Wilms tumour. Ultrasound provides real-time visualisation of the tumour boundaries, enabling the surgeon to precisely delineate tumour anatomy during resection. The integration of intraoperative cross-sectional imaging into the surgical workflow, however, remains complex due to challenges in maintaining real-time dynamic imaging without disrupting the surgical process. Nevertheless, high-resolution ultrasound and other imaging technologies have the potential to expand the scope and effectiveness of minimally invasive paediatric cancer surgery.

While the application of these advanced technologies in paediatric cancer surgery is promising, several challenges remain. The integration of new technologies into clinical practice requires substantial investment in equipment and training. Additionally, long-term studies are needed to establish the safety, efficacy and cost-effectiveness of these techniques. Robotic surgery, single-site approaches and image-guided surgery can potentially reduce morbidity and improve surgical outcomes; however, evidence supporting these advantages yet remains of very low quality. Moreover, evidence examining the long-term impact of these techniques on overall survival and recurrence rates in paediatric cancer patients is lacking.

As these technologies continue to evolve, it will be essential to ensure that they are accessible to all paediatric cancer patients, regardless of socioeconomic status. Ethical considerations, including cost, access and informed consent, must also be addressed as these technologies become more integrated into clinical practice.

## Conclusion

The integration of robotic surgery, single-site techniques and advanced image guidance into minimally invasive paediatric cancer surgery marks a significant advancement in the field. These technologies hold the potential to enhance surgical precision, reduce patient morbidity and improve the overall quality of life for patients. However, to fully realise their benefits, ongoing research, collaboration and innovation among clinicians, researchers and technologists are essential. This will ensure that these techniques are refined, standardised and implemented safely across paediatric oncology surgery. As the field continues to evolve, the combination of cutting-edge technology with expert surgical care will pave the way for a new era of precision surgery—one that offers more effective, less invasive treatments with better outcomes for paediatric cancer patients.

## Conflicts of interest

The authors declare that there are no conflicts of interest related to this work.

## Funding

This research received no specific grant from any funding agency in the public, commercial or not-for-profit sectors.
